# Improvement of Biomass and Phycoerythrin Production by a Strain of *Rhodomonas* sp. Isolated from the Tunisian Coast of Sidi Mansour

**DOI:** 10.3390/biom12070885

**Published:** 2022-06-24

**Authors:** Hana Derbel, Jihen Elleuch, Latifa Tounsi, Marco Sebastiano Nicolo, Maria Giovanna Rizzo, Philippe Michaud, Imen Fendri, Slim Abdelkafi

**Affiliations:** 1 Laboratoire de Génie Enzymatique et Microbiologie, Equipe de Biotechnologie des Algues, Ecole Nationale d’Ingénieurs de Sfax, University of Sfax, 3038 Sfax, Tunisia; hana.derbel@enis.tn (H.D.); jihen.elleuch@enis.tn (J.E.); latifa.tounsi@enis.tn (L.T.); 2 Department of Chemical, Biological, Pharmaceutical and Environmental Sciences, University of Messina, Viale F. Stagno d’Alcontres, 31, 98166 Messina, Italy; mnicolo@unime.it (M.S.N.); mariagiovanna.rizzo@unime.it (M.G.R.); 3 Institut Pascal, Université Clermont Auvergne, CNRS, Clermont Auvergne INP, 63178 Clermont-Ferrand, France; 4 Laboratory of Plant Biotechnology, Faculty of Sciences of Sfax, University of Sfax, 3038 Sfax, Tunisia; imen.fendri@fss.usf.tn

**Keywords:** *Rhodomonas* sp., phycoerythrin, microalgae, *cpeB* gene

## Abstract

Microalgae are photoautotrophic microorganisms known as producers of a large variety of metabolites. The taxonomic diversity of these microorganisms has been poorly explored. In this study, a newly isolated strain was identified based on the 18S rRNA encoding gene. The phylogenetic analysis showed that the isolated strain was affiliated with the *Rhodomonas* genus. This genus has greatly attracted scientific attention according to its capacity to produce a large variety of metabolites, including phycoerythrin. Growth and phycoerythrin production conditions were optimized using a Plackett–Burman design and response surface methodology. An expression profile analysis of the *cpeB* gene, encoding the beta subunit of phycoerythrin, was performed by qRT-PCR under standard and optimized culture conditions. The optimization process showed that maximum cell abundance was achieved under the following conditions: CaCl_2_ = 2.1328 g/L, metal solution = 1 mL/L, pH = 7 and light intensity = 145 μmol photons/m^2^/s, whereas maximum phycoerythrin production level occurred when CaCl_2_ = 1.8467 g/L, metal solution = 1 mL/L, pH = 7 and light intensity = 157 μmol/m^2^/s. In agreement, positive transcriptional regulation of the *cpeB* gene was demonstrated using qRT-PCR. This study showed the successful optimization of abiotic conditions for highest growth and phycoerythrin production, making *Rhodomonas* sp. suitable for several biotechnological applications.

## 1. Introduction

Microalgae are photosynthetic organisms classified based on their color into four groups, namely, red, green, blue-green and brown microalgae. They are able to synthesize a large variety of metabolites such as proteins, lipids, carbohydrates and pigments [1,2,3,4,5]. These compounds may serve in several biotechnological fields including food, cosmetics, as well as the biomedical and pharmaceutical sectors [6,7,8,9].

Among these metabolites, phycobiliproteins (PBPs) are the major pigments of red algae, Cyanobacteria and Cryptophyta. They have attracted great attention from the scientific community thanks to their spectral, fluorescent and colorant properties [10]. These chromoproteins are light-harvesting compounds absorbing in a spectral region different to that of chlorophylls [11,12]. Phycobiliproteins can be classified into phycoerythrins (PEs; λ_max_ = 540–570 nm), phycocyanins (PCs; λ_max_ = 610–620 nm), allophycocyanins (APCs; λ_max_ = 650–655 nm) and phycoerythrocyanins (PECs; λ_max_ = 560–600 nm) [13,14].

In red algae (Rhodophyta) and Cyanobacteria, PBPs are organized into phycobilisomes, but in cryptomonads, the phycobiliprotein antenna is usually unique and located in the thylakoid lumen [15,16,17,18]. Phycoerythrins are the major light harvesting pigments for Cryptophyta species [1]. They can be used in several biotechnological applications, including food, cosmetic, immunochemistry, clinical diagnostics, biological engineering as well as the therapeutic field [7,8,19,20]. Thus, the enhancement of PE biosynthesis, as well as an understanding of its regulatory pathway, are of interest. Cryptophyta microalgae belonging to *Rhodomonas* genus are known as potential candidates for PE production, as this pigment represent about 51% of total pigments produced by some strains of this genus [21]. Many abiotic factors affect PE production by *Rhodomonas* genus, including temperature, nutrient concentrations, light quality (wavelength) and intensity [22,23,24]. In fact, high temperature and nitrate concentration combined with low light intensity enhanced PE content in *R. salina* [22]. The crystal structure of phycoerythrin from the marine cryptophyte *Rhodomonas* has been determined [25]. It is an α1α2ββ heterodimeric protein encoded by the *cpeB cpeA* operon that is partially regulated by light quality [11,13,26].

The aim of this work was first to optimize the culture conditions for providing high phycoerythrin production by a newly isolated microalga, *Rhodomonas* sp., and then to investigate the phycoerythrin production profile under optimal and standard experimental conditions, with a corresponding transcriptome analysis.

## 2. Materials and Methods

### 2.1. Isolation of Axenic Monoclonal Cultures

Microalgae cells were isolated from the Tunisian Coast of Sidi Mansour (governorate of Sfax, Mediterranean Sea) (Latitude: 34°47′41.8″ N; Longitude: 10°51′06.9″ E) in January 2018. Water samples were collected from the sampling site using a plastic bucket. The collected seawater samples were loaded into a sterile transparent glass jar and transported in an isotherm icebox to the Biotechnology Unit of Algae (UBA), where they were immediately processed. Seawater samples were subjected to successive filtration. Firstly, a 60 µm pore size membrane was used to eliminate protozoa. Then, the obtained filtrates underwent filtration with a 2.5 μm pore size membrane to remove bacteria. Each membrane was divided afterwards into two portions: the first portion was flooded with 100 mL of F/2 Provasoli medium [27] (Supplementary Table S1) and the second was immersed into 50 mL of Pm medium (Supplementary Table S1) [27,28]. Algal growth was monitored using an inverted microscope (Motic microscope AE 2000, Barcelona, Spain). Pure cultures were obtained by micromanipulation after serial dilutions and successive plating on agar media [4]. The obtained isolated strain was maintained in F/2 medium at 23 ± 2 °C continuously illuminated by white light with an intensity irradiance of approximately 80 µmol/m^2^/s. The algae density was measured daily by measuring optical density (OD) at 550 nm and cell counting using a Malassez counting chamber and an inverted microscope (Motic microscope AE 2000, Barcelona, Spain).

### 2.2. DNA Extraction, PCR Amplification, Sequencing and Phylogenetic Analysis

Genomic DNA from the newly isolated strain was extracted using Pure link Genomic DNA Mini kit (Invitrogen, Waltham, MA, USA) according to the manufacturer’s protocol. Two universal primers were used for the PCR reaction to amplify the 18S rRNA gene (Table 1) [29]. PCR was performed as described by Fendri et al. [30]. After purification of the PCR products, sequencing with EukA and EukB primers was performed [31]. The obtained sequences were compared with the sequences available in GenBank using the BLAST server from the NCBI website (http://www.ncbi.nlm.nih.gov/BLAST; accessed on 1 June 2019).

### 2.3. Growth and Phycoerythrin Accumulation

Five growth media were tested for isolated microalgae growth: BG-11 [32], standard and modified F/2 [27], Pm [33] and sterilized artificial seawater (Supplementary Table S1).

PE production was monitored every 48 h for 21 days. PE extraction was performed according to the method reported by Gargouch et al. [20]. Two mL of microalgal culture were centrifuged at 7200× *g* for 10 min. Pellets were suspended in 1 mL of sodium phosphate buffer (0.1 M, pH 6.0) and cell lysis was performed with a combination of repetitive freeze-thaw cycles followed by sonication for 10 min. Samples were maintained at 4 °C for 4 h to ensure maximum extraction of pigments [34] and centrifuged at 10,000× *g* for 30 min at 4 °C. Supernatants were collected and PE concentration was calculated according to Lawrenz et al. [34] using Equation (1):

PE (µg/L) = (A_545_ − A_750_)/ɛd × Mw × V_sample_/V_buffer_ × 10^6^ (1)

where ε and Mw are the molar extinction coefficients (2.41 × 10^6^ L/mol/cm) and molecular weight of the phycoerythrin (240,000 g/mol), respectively, d is the path length of the cuvette, and V_sample_ and V_buffer_ are the volume of the sample and buffer, respectively.

### 2.4. Experimental Design and Data Analysis

#### 2.4.1. Screening of Factors Influencing Phycoerythrin Production

In order to screen the main factors affecting the production of PE, a Plackett–Burman statistical experimental design was performed. Thirteen variables were chosen, corresponding to all modified F/2 medium components as well as pH and light intensity. Each independent variable was investigated at a high (+1) and a low (−1) level based on bibliographic data. Table 2 presents all factors and the tested levels. 

Each experiment was performed in 250 mL Erlenmeyer flasks containing 100 mL culture media and inoculated with the exponentially growing microalgae culture at 10% (*v*/*v*). All experiments were performed in duplicate and PE production level was assessed after 16 days of incubation at 25 °C. A Plackett–Burman design was carried out using STATISTICA software 12.0 (Stat Soft. Inc., 1984–2014, Tulsa, OK, USA). Plackett–Burman data analysis allowed the identification of factors that had a significant effect, either positively or negatively, on PE production.

#### 2.4.2. Optimization of Growth and Phycoerythrin Production Using a Box–Behnken Model

A Box–Behnken design was employed to study the interaction effects between four significant variables identified by Plackett–Burman design to determine their optimal levels. Based on the results of a preliminary experiment, the ranges of the variables were chosen (Table 3). A total of 27 trials were performed, and the independent variables were investigated at three different levels (−1, 0 and +1). All experiments were performed in duplicate, as described previously (Section 2.4.1.). The amount of PE (Y_PE_) and biomass abundance (Y_Biomass_) were determined after 16 days of incubation at 25 °C and compared for each combination of the independent variables. The amount of PE was determined by the method mentioned above. Biomass abundance was determined as previously described [35]. Briefly, cells were harvested by centrifugation at 10,000× *g* for 10 min and washed twice with sterile distilled water. The obtained fresh biomass was then oven-dried at 105 °C until a constant weight.

Subsequently, response surface methodology (RSM) was used to determine their optimal levels and to study individual and mutual interactions among the tested variables [36]. A second-degree polynomial equation was used to calculate the relationship between the independent variables and the design experiment responses, Y_PE_ and Y_Biomass_. Given all linear, square and interaction coefficients, the quadratic regression model can be illustrated as follows:

Y = β_0_ + Σ β_i_X_i_ + Σ β_ij_X_i_X_j_ + Σβ_ii_X_i_^2^ (2)

where Y is the predicted response; β_0_ is the intercept term; β_i_ is the linear coefficient; β_ii_ is the quadratic coefficient; β_ij_ is the interaction coefficient. X_i_ and X_j_ are the levels of the independent variables. The dependent response value was the mean of two independent experiments. The independent variables and the dependent output responses were modelled using an analysis of variance (ANOVA) to justify the adequacy of the models using Nemrod-W^®^ software (LPRAI, Marseille, France). The chosen confidence interval was of the order of 95%, corresponding to *p* < 0.05.

### 2.5. Gene Expression Analysis

Day 15-aged cells from standard and optimized cultures were harvested by centrifugation at 10,000× *g* for 10 min at 4 °C and used for total RNA extraction using TRIzol reagent (Invitrogen, Carlsbad, CA, USA) according to the protocol adapted from Meng and Feldman [37]. Briefly, pellets were suspended in 1 mL of TRIzol reagent and vortexed until total dissolution. Then, 200 µL of chloroform was added and the mixtures were incubated for 2 min and then centrifuged at 12,000× *g* at 4 °C for 15 min. The upper aqueous phases were recovered and mixed with isopropyl alcohol and then incubated for 10 min. After centrifugation at 12,000× *g* at 4 °C for 10 min, pellets were dissolved with 75% ethanol and incubated overnight at −20 °C. The mixtures were then centrifuged at 7500× *g* at 4 °C for 5 min and the pellets were dried. Total RNA was then dissolved in RNase-free water and stored at −80 °C. The resulting total RNA was analyzed using agarose gel electrophoresis (2%) [38] and its purity and quantity were determined spectrometrically using a NanoDrop 2000 spectrometer (Thermo Scientific, Waltham, MA, United States).

One µg of each RNA sample was used for cDNA synthesis using PrimeScript™ RT Reagent Kit with gDNA Eraser (Perfect Real Time) (Takara, Kyoto, Japan) according to the manufacturer’s procedure. Real-time q-PCR was conducted for *cpeB* gene (Table 1). All q-PCR assays were carried out on a StepOnePlus™ PCR cycler (Applied Biosystems, Foster City, CA, USA). Amplification reactions were performed in a 20-μL reaction mix containing 10 μL of TB Green Premix Ex Taq II (Tli RNase H Plus) (2x) (Takara, Kyoto, Japan), 1 μM of each primer (Bio Basic Canada Inc, Markham, Canada), and 3 μL of each appropriate cDNA sample. The used cycling conditions were 30 s at 95 °C, followed by 40 cycles of 15 s at 95 °C and 1 min at 60 °C. At the end of the q-PCR cycles, the amplification specificity of each primer pair was verified with a fusion step performed by heating from 60 °C to 95 °C. All experiments were run in triplicate. The α-tubulin gene was used as a housekeeping gene (Table 1). Reactions with an amplification efficiency between 95% and 105% were considered acceptable [39,40,41]. Relative mRNA expression values were determined with the 2-^ΔΔCt^ method [42,43].

### 2.6. Statistical Analysis

The occurrence of statistically significant differences in the analyzed data was determined using a one-way ANOVA followed by Duncan multiple range tests. Differences were considered significant at *p* < 0.05. All analyses were carried out using SPSS 17.0 software (SPSS, Inc., Chicago, IL, USA). All measurements were made in triplicate and the results were expressed as means.

## 3. Results

### 3.1. Isolation and Identification of the Microalgae Strain

A microalgae strain was isolated from the Sfax coast and identified as belonging to the Cryptophyceae class based on morphometric characteristics using light microscopy. The 18S rRNA coding gene from the newly isolated strain was successfully amplified and partially sequenced using universal primers (Table 1) (Supplementary Table S2). The BLASTn results for the obtained 18S rDNA sequence showed 88.89% identity with the *Rhodomonas* genus.

### 3.2. Screening for Culture Media Suitable for Cell Growth and Phycoerythrin Production

**Figure 1:**
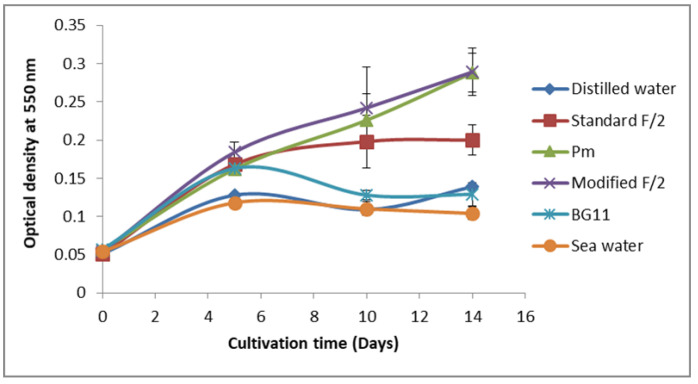
Screening of the most suitable culture media for *Rhodomonas* sp. growth. (Data are means ± SE).

**Figure 2:**
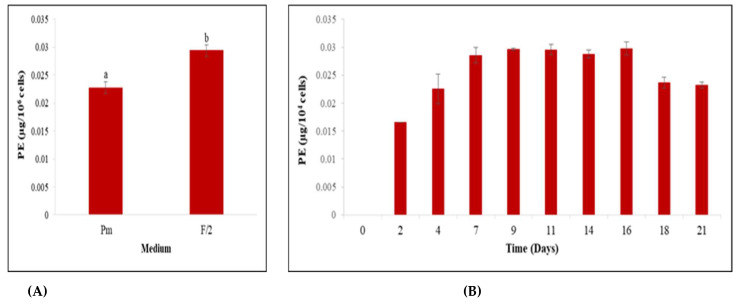
PE production capacity by *Rhodomonas* sp. strain. (**A**) Comparison of modified F/2 and Pm medium; (**B**) time-course using modified F/2. (Data are means ± SE. Columns labeled with different low case letters (a, b) indicate statistically significant differences (*p* < 0.05, ANOVA, Duncan multiple range tests)).

Different culture media with diverse chemical compositions were tested to select the best growth medium and the most suitable one for PE production (Figure 1 and Figure 2).

Data presented in Figure 1 showed that the maximum growth of *Rhodomonas* sp. was supported by modified F/2 and Pm media. Thus, PE production by *Rhodomonas* sp. was then evaluated for these two media.

Regarding PE production, the obtained results (Figure 2A) demonstrated that the PE yield obtained from *Rhodomonas* sp. cells was significantly better when cultured in modified F/2 medium than Pm medium (*p* < 0.05). Therefore, modified F/2 was selected for the rest of this study.

To determine the optimum time required for PE production by *Rhodomonas* sp. cells, both cell growth and PE production rates in accordance with time were taken into consideration. The obtained results, summarized in Figure 2B, showed that *Rhodomonas* sp. reached the highest PE content of 0.29 µg/10^4^ cells on day 16, which coincides with the stationary phase.

### 3.3. Screening of Factors Influencing Phycoerythrin Production

A Plackett–Burman design was used to evaluate the effects of all components of modified F/2 medium, as well as pH and light intensity, on phycoerythrin production. Table 4 illustrates the statistical significance of the variables, as determined by Student’s t-test.

Results from the Plackett–Burman design revealed that CaCl_2_ concentration (X_3_), metal solution (X_10_), light intensity (X_12_) and pH (X_13_) were the most significant factors affecting PE production, and thus, were selected for further optimization using Box–Behnken design. As shown in Table 5, metal solution and pH exerted a negative effect on PE production, whereas the PE rate reached higher values with high light intensities and CaCl_2_ concentrations. Other low significant and insignificant variables were maintained in all subsequent trials according to their concentration in modified F/2 medium for further optimization by Box-Behnken design.

### 3.4. Combined Effects of Modified F/2 Medium Components on Biomass and Phycoerythrin Production

After selecting the most significant factors for PE production based on the data obtained from the Plackett–Burman plan, a Box–Behnken design was used in order to accomplish the optimization of the selected variables by studying their effects on PE production as well as biomass accumulation. Table 3 summarizes the variables and experimental levels used for optimizing culture conditions. Twenty-seven experiments were carried out, and the relationship between the independent variables and both responses (PE production and biomass accumulation) were determined by multiple-regression statistical analysis. The regression variables for the predicted response surface quadratic model are presented in Table 6 and Table 7.

An ANOVA was conducted to test the significance of the fit of the second-order polynomial equation to the experimental data, as given in Table 8 and Table 9. Statistical significance of the variables and their interactions at various levels of probability are depicted based on a distribution test and analysis of variance fitted to second-order polynomial Equation (2). The most significant corresponding coefficients were selected. The obtained results revealed linear and quadratic effects of light intensity, X_4_ and X_4_^2^, respectively, and the interaction between light intensity and CaCl_2_ had a highly positive significant effect on the production of PE (Table 8).

Regarding biomass accumulation, it was found that CaCl_2_ concentration (X_1_) as well as light intensity (X_4_) had highly positive significant effects. The quadratic significant effect of pH (X_3_^2^) was identified as negative, whereas CaCl_2_ concentration (X_1_ X_4_) presented a positive effect on biomass production (Table 9).

Therefore, Equation (2) was rearranged to Equations (3) and (4).

Y_PE_ = 0.8115 + 0.7468 X_4_ + 0.4625 X_4_^2^ + 0.7845 X_1_X_4_ (3)

Y_Biomass_ = 283.33333 + 67.70833 X_1_ + 120.87500 X_4_ − 79.72917 X_3_^2^ + 96.87500 X_1_X_4_ (4)

where Y_PE_ and Y_Biomass_ represent the predicted PE production level and biomass accumulation, respectively. The obtained results showed a non-significant value for the lack of fit (*p* > 0.05) as well as highly significant R*^2^* values of 0.86 and 0.83 related to PE production and biomass accumulation, respectively.

**Figure 3:**
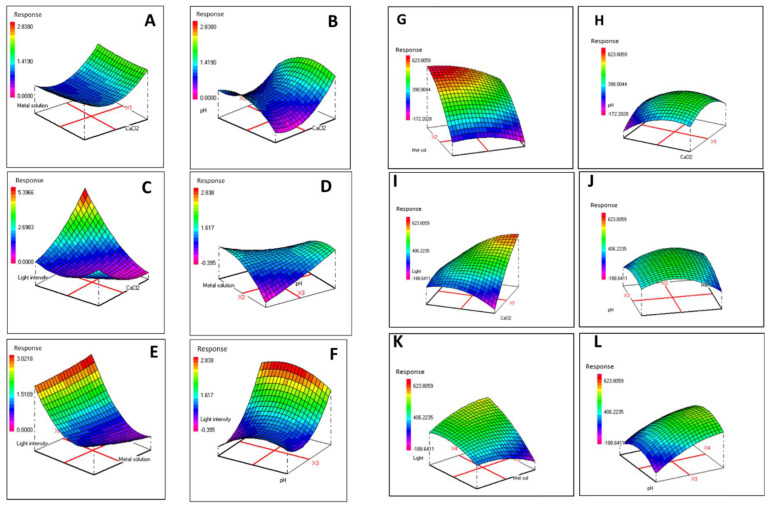
Response surface plots for PE production (**A**-**F**) and biomass accumulation (**G**-**L**) as a function of the different studied variables: (**A**) metal solution and CaCl_2_ concentration; (**B**) CaCl_2_ concentration and pH; (**C**) light intensity and CaCl_2_ concentration; (**D**) pH and metal solution; (**E**) metal solution and light intensity; (**F**) light intensity and pH; (**G**) metal solution and CaCl_2_ concentration; (**H**) CaCl_2_ concentration and pH; (**I**) light intensity and CaCl_2_ concentration; (**J**) pH and metal solution; (**K**) metal solution and light intensity; (**L**) light intensity and pH.

Response surface methodology (RSM) was then used to examine the effect of interactions between the tested variables on PE production and biomass accumulation. Three dimensional (3D) plots were used to visualize the relationship between two parameters while the other parameters were held constant (Figure 3). The effects of light intensity and CaCl_2_ concentration on PE production and biomass accumulation are shown in Figure 3C.I, respectively. PE production clearly increased as light intensity and CaCl_2_ concentration increased. Similar results were obtained for biomass accumulation.

Accordingly, optimized PE production (5.36 ± 0.68% of DW) was obtained under the following conditions: CaCl_2_ = 1.8467 g/L, metal solution = 1 mL/L, pH = 7 and light intensity = 157 μmol/m^2^/s. Maximum biomass accumulation (633.33 ± 57.73 mg/L) was achieved using 2.1328 g/L CaCl_2_, 1 mL/L of metal solution, pH 7 and 145 μmol/m^2^/s light intensity.

By comparison, PE production and biomass accumulation under standard conditions (1.08 ± 0.04% of DW and 333.33 ± 28.86 mg/L, respectively) were enhanced by about 5- and 2-fold, respectively, using the optimized conditions.

3.5. cpeB Gene Expression Profile under Optimal Conditions

**Figure 4:**
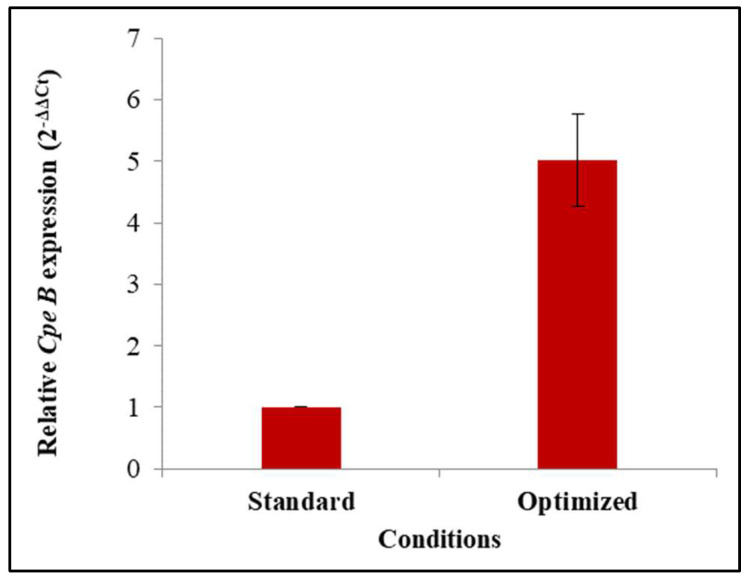
*cpeB* gene expression levels for *Rhodomonas* sp. using different culture conditions.

Due to the capacity of *Rhodomonas* sp. to produce PEs [23], an understanding of regulatory mechanisms involved in its synthesis is of great interest. Therefore, the expression level of the *cpeB* gene was investigated. Reverse transcription (RT) followed by the real-time polymerase chain reaction was used to analyze mRNA expression in microalgae cells cultured under optimized and standard conditions. To investigate physiological changes in *cpeB* gene expression, the relative expression of *cpeB* target gene versus the α-tubulin reference gene was determined. The obtained results showed that the expression level of the *cpeB* gene was up-regulated by approximately 5-fold by the optimal conditions for PE production compared to the control (Figure 4). Thus, modulation of the expression of the *cpeB* gene, coding for a PE subunit, proves the existence of a transcriptional regulatory mechanism.

## 4. Discussion

Thanks to their various interesting biotechnological applications in food, pharmaceuticals, and cosmetics industries, phycobiliproteins attract the attention of researchers and consumers. However, market development of this pigment is dependent on limiting the costs for large-scale production. Cryptophyte microalgae are one possible source of phycobiliproteins, especially PE. Members of the *Rhodomonas* genus, belonging to Cryptophyte microalgae, are typically found in marine environments, although freshwater strains have been reported [42], and are known to be good producers of PE. In the present study, a *Rhodomonas* sp. strain has been isolated from the local marine environment showing potential for PE production. The monitoring of PE production showed that the highest PE yield was reached on day 16 of culture, which correspond to the stationary phase. This result is in concordance with previous data reported by Marraskuranto et al. in 2018 [44]. Many publications support that abiotic factors have substantial effects on biomass and pigments production. It has been shown that the amount of PE accumulating in *Rhodomonas* sp. depends on cultivation conditions [24,45]. Thus, an experimental screening of the factors that could impact the production of PE was performed using a Plackett–Burman design. The obtained results revealed that CaCl_2_ concentration, metal solution, pH and light intensity were the most important PE production variables. It has previously been reported that PE production by *R. salina* could be affected by a variety of abiotic factors, particularly temperature, nutrients, light quality and intensity [22,23,24].

After selecting the most significant factors for PE production, a Box–Behnken design was used in order to accomplish the optimization of the selected factors by studying their effects as well as their interactions. Regarding the optimization of PE content, CaCl_2_ concentration and light intensity were the two main factors impacting PE production by *Rhodomonas* sp. (Figure 3). In fact, the highest PE yield (5.36 ± 0.68% of DW) was achieved by increasing the light intensity and CaCl_2_ concentration to 157 μmol/m^2^/s and 1.8467 g/L, respectively, and fixing the metal solution and pH factors at 1 mL/L and 7, respectively. Interestingly, PE synthesis increased significantly under optimized conditions by approximately 5-fold compared to the control. In concordance, Chaloub et al. [12] and Xie et al. [14] reported that PE production by *Rhodomonas* genus depended on light quality and quantity [14,24]; however, *R. salina* culture at pH 8.5 and 40% salinity also increased phycoerythrin productivity [46]. It is important to note that these studies were performed for a shorter culture duration (between 4 and 10 days) compared to the present study. Xie et al. [14] evaluated PE production by *R. salina* cultured for different times and under different light intensities. They demonstrated that PE concentration during long culture periods was enhanced as the light intensity increased, which was due to the accumulation of high cell densities at these light intensities [14,24]. This finding can explain the higher optimal value for light intensity found in the present study compared to previous studies [12,44].

The highest biomass productivity (633.33 ± 57.73 mg/L) was obtained with a light intensity and CaCl_2_ concentration of about 145.5 μmol/m^2^/s and 2.1328 g/L, respectively. In concordance, Oostlander et al. [46] reported an increase in biomass yield for *Rhodomonas* sp. strain obtained with light intensities ranging from 110 to 220 μmol/m^2^/s. High biomass productivity was previously observed under continuous green light illumination conditions [24] and increasing calcium concentration had a positive impact on algae biomass production [47].

A transcriptomic-based analysis of the *cpeB* gene expression for *Rhodomonas* sp. under different growth conditions was conducted. In fact, the expression level of the *cpeB* gene, encoding the beta subunit of phycoerythrin, was investigated in microalgae cells cultivated under standard and optimized conditions using qRT-PCR. The expression profile of *cpeB* gene was significantly up-regulated by the optimal conditions for PE production compared to the control. This result suggests the existence of a transcriptional regulation mechanism for the studied gene. There are few data available concerning the control and regulation of PE production [48]. The expression of photosynthetic related genes is regulated in response to environmental conditions and controlled at the transcriptional and post-transcriptional levels in algae and cyanobacteria [49,50]. He et al. [51] reported that under high light irradiance, the expression of genes involved in carotenoid biosynthesis, as well as the genes related to photosynthesis-antenna proteins, fatty acid elongation and carbon fixation, was significantly changed in photosynthetic organisms. Furthermore, several studies found that PE and PC synthesis in red algae were affected by both irradiance and spectral composition [24,52,53,54]. Another study suggested that the expression of genes involved in the pathway for photosynthesis-antenna proteins could be inhibited by acetate addition [51]. In addition, Thien et al. [55] found that under CO_2_ enrichment, the expressions of genes related to phycobilisome, PE and photosystem proteins were upregulated in *Kappaphycus alvarezii* (Rhodophyta).

## 5. Conclusions

An isolated microalga strain was identified as belonging to the *Rhodomonas* genus based on 18S rRNA gene sequencing. Plackett–Burman and Box–Behnken designs were used to maximize PE production and biomass accumulation in this strain. Following the optimization, the production of biomass and PE were enhanced by 2- and 5-fold, respectively, compared to standard conditions. Therefore, the high potential for the production of PE revealed by *Rhodomonas* sp. makes this strain a promising candidate for various biotechnological and industrial applications. In concordance, a transcriptomic study revealed that the *cpeB* gene was over-expressed under optimized conditions compared to standard culture conditions. This provides a better understanding about the metabolic pathway of the PE biosynthesis in *Rhodomonas* genus.

## Figures and Tables

**Table 1:** List of primers used.

Gene Abbreviation	Description	Primer Sequence ^1^
α-tub	α-tubulin	F: 5′-AGATCACGAACGCCACCTTC-3′
		R: 5′-GATGGTGCGCTTCGTCTTGA -3′
*cpeB*	phycoerythrin beta chain	F: 5′-GGTGGTGCAGATCTACAAGC-3′
		R: 5′-CATGCAGCCATACGACGGTT-3′
18S	Ribosomal RNA 18 S	F: 5′-AACCTGGTTGATCCTGCCAGT-3′
		R: 5′-TGATCCTTCTGCAGGTTCACCTAC-3′

^1^ F = forward; R = reverse.

**Table 2:** Plackett–Burman experimental design for the evaluation of 13 independent variables on PE production.

Coded Factors	Factors	Low Level (−1)	High Level (+1)
X_1_	NaCl (g/L)	17	37
X_2_	MgCl_2_ (g/L)	2	9
X_3_	CaCl_2_ (g/L)	0	2
X_4_	KNO_3_ (g/L)	0	2
X_5_	KH_2_PO_4_ (g/L)	0	0.12
X_6_	NaHCO_3_ (g/L)	0	0.1
X_7_	MgSO_4_ (g/L)	1	5.5
X_8_	NaNO_3_ (g/L)	0.5	1.5
X_9_	NaH_2_PO_4_ (g/L)	0.5	1.5
X_10_	Metal Solution (mL/L)	0.5	1.5
X_11_	Vitamin Solution (mL/L)	0	1
X_12_	Light intensity (µmol/m^2^/s)	27	110
X_13_	pH	5	9

**Table 3:** Variables and experimental levels for optimizing culture conditions.

Coded Factors	Factors	−1	0	+1
X_1_	CaCl_2_ (g/L)	0.25	1.125	2
X_2_	Metal solution (mL/L)	0.5	1	1.5
X_3_	pH	5	7	9
X_4_	Light intensity (µmol/m^2^/s)	43	92	141

* −1, 0 and 1 correspond to the minimum, medium and maximum of the input variable range, respectively.

**Table 4:** Analysis of variance for the phycoerythrin response.

Source of Variation	DF	Seq SS	Adj SS	Adj MS	F-Value	*p*-Value
Main Effects	13	3129.08	3129.08	240.698	6.95	0.000
Residual Error	26	900.06	900.06	34.618		
Lack of fit	6	868.05	868.05	144.675	90.39	0.000
Pure error	20	32.01	32.01	1.601		
Total	39	4029.14				

**Table 5:** Effect of different operational variables on PE production.

Terms	Coefficient	*p*-Value
Constant	4.565	0.000
NaCl	−2.612	0.009 *
MgCl_2_	−2.026	0.039 *
CaCl_2_	4.224	0.000 **
KNO_3_	2.313	0.020 *
KH_2_PO_4_	−0.486	0.606
NaHCO_3_	1.535	0.111
MgSO_4_	1.668	0.085
NaNO_3_	−0.637	0.500
NaH_2_PO_4_	−1.693	0.080
Metal solution	−3.430	0.001 **
Vitamin solution	−0.877	0.355
Light intensity	3.396	0.001 **
pH	−3.376	0.001 **

* Significant at level 95%, ** Significant at level 99%.

**Table 6:** Analysis of variance for phycoerythrin production response.

Source of Variation	Sum Squares	Degrees of Freedom	Mean Square	Ratio	Significance (%)
Regression	3.4357	14	0.9597	5.0189	**
Residual	2.1034	11	0.1912		
Lack of fit	2.0855	10	0.2086	11.6767	22.8%
Error	0.0179	1	0.0179		
Total	5.5391	25			

** Significant at 99%.

**Table 7:** Analysis of variance for biomass production response.

Source of Variation	Sum of Squares	Degrees of Freedom	Mean Square	Ratio	Significance (%)
Regression	3.27605E + 0005	14	2.34003E + 0004	4.3819	**
Residual	6.40829E + 0004	12	5.34024E + 0003		
Lack of fit	5.33538E + 0004	10	5.33538E + 0003	0.9946	59.8%
Error	1.07291E + 0004	2	5.36458E + 0003		
Total	3.91688E + 0005	26			

** Significant at 99%.

**Table 8:** Statistical analysis of the coefficients for PE production.

Factor	Coefficient	F. Inflation	Ecart-Type	t.exp.	Significance. %
X_0_	0.8115		0.3092	2.62	*
X_1_	0.2565	1.00	0.1262	2.03	6.5%
X_2_	−0.0699	1.00	0.1262	−0.55	59.6%
X_3_	0.0367	1.00	0.1262	0.29	77.3%
X_4_	0.7468	1.00	0.1262	5.92	***
X_1_^2^	0.3367	1.48	0.2093	1.61	13.3%
X_2_^2^	0.0358	1.48	0.2093	0.17	86.2%
X_3_^2^	−0.2579	1.48	0.2093	−1.23	24.3%
X_4_^2^	0.4625	1.48	0.2093	2.21	*
X_1_X_2_	0.0445	1.00	0.2186	0.20	83.6%
X_1_X_3_	−0.1605	1.00	0.2186	−0.73	48.4%
X_2_X_3_	−0.3578	1.00	0.2186	−1.64	12.7%
X_1_X_4_	0.7845	1.00	0.2186	3.59	**
X_2_X_4_	0.1285	1.00	0.2186	0.59	57.4%

*** Significant at 99.9%, ** Significant at 99%, * Significant at 95%.

**Table 9:** Statistical analysis of the coefficients for PE production response.

Factor	Coefficient	F. Inflation	Ecart-Type	t.exp.	Significance (%)
X_0_	283.33333		42.19101	6.72	***
X_1_	67.70833	1.00	21.09550	3.21	**
X_2_	1.04167	1.00	21.09550	0.05	96.0%
X_3_	6.29167	1.00	21.09550	0.30	76.7%
X_4_	120.87500	1.00	21.09550	5.73	***
X_1_^2^	−62.47917	1.25	31.64325	−1.97	6.9%
X_2_^2^	−34.35417	1.25	31.64325	−1.09	30.0%
X_3_^2^	−79.72917	1.25	31.64325	−2.52	*
X_4_^2^	−39.10417	1.25	31.64325	−1.24	23.9%
X_1_X_2_	21.87500	1.00	36.53848	0.60	56.7%
X_1_X_3_	−15.62500	1.00	36.53848	−0.43	67.9%
X_2_X_3_	−28.12500	1.00	36.53848	−0.77	46.2%
X_1_X_4_	96.87500	1.00	36.53848	2.65	*
X_2_X_4_	53.12500	1.00	36.53848	1.45	16.9%

*** Significant at 99.9%, ** Significant at 99%, * Significant at 95%.

## Data Availability

Not applicable.

## Supplementary Materials

The following supporting information can be downloaded at: https://www.mdpi.com/article/10.3390/biom12070885/s1, Table S1: Nutrients composition of the different growth media used; Table S2: Partial sequence of 18S rRNA gene of the isolated Rhodomonas sp.
