# Supplementary material for: Improvement of Biomass and Phycoerythrin Production by a Strain of Rhodomonas sp. Isolated from the Tunisian Coast of Sidi Mansour
Source: Biomolecules. 2022 Jun 24;12(7):885. doi: 10.3390/biom12070885 (PMC9312907; doi:10.3390/biom12070885)
Supplement: Supplementary file 1 [file biomolecules-12-00885-s001.zip › biomolecules-1721990-supplementary.pdf]

**Table S1.** Nutrients composition of the different growth media used.

| Culture Media | Compositions | References |
| --- | --- | --- |
| **Pm medium** | 15 g/L NaCl, 1.7 g/L $NaNO_3$, 0.9 g/L $K_2HPO_4$, 7.2 g/L $MgSO_4$, $7H_2O$, 1.55 g/L $CaCl_2$, $2H_2O$ and 1 mL/L trace metal solution composed of 0.15 g/L $MnSO_4$, $H_2O$, 0.015 g/L $(NH_4)6Mo_7O_{24}$, $4H_2O$, 0.015 g/L $CoSO_4$, $7H_2O$, 0.05 g/L $CuSO_4$, $5H_2O$, 0.25 g/L $ZnSO_4$, $7H_2O$ and 10 g/L Fer-EDTA and 1 mL/L of vitamin solution prepared in pure water and containing 0.01 g/L Vitamin B12, 0.025 g/L Thiamin and 0.04 g/L Biotin | Soanen N, Da Silva E, Gardarin C, Michaud P, Laroche C. Improvement of exopolysaccharide production by *Porphyridium marinum*. Bioresour Technol. 2016 Aug;213:231-238. doi: 10.1016/j.biortech.2016.02.075. Epub 2016 Feb 26. PMID: 26944455. |
| **Artificial sea water** | 24.53g/L of NaCl; 5.2 g/L of $MgCl_2$; ;4.09 g/L of$Na_2SO_4$;1.54 g/L of $CaCl_2$, $2H_2O$; 0.695 g/L of KCL; 0.2g/L of $NaHCO_3$; 0.101 g/L of KBr; 0.027 g/L of $H_3BO_3$;0.42 g/L of SrCl2, $6H_2O$; 0.03 g/L of NaF. | Yeh, C. P., Tsai, K. C., & Huang, J. Y. (2020) Influence of Chloride Concentration on Stress Corrosion Cracking and Crevice Corrosion of Austenitic Stainless Steel in Saline Environments. Materials (Basel, Switzerland), 13(24), 5640. https://doi.org/10.3390/ma13245640 |
| **Standard F/2 medium** | 150 mg of $NaNO_3$; 10 mg of $NaH_2PO_4$, $H_2O$; 10 mg of Fe sequestrene; 30-60 mg of $Na_2SiO_4$, $9H_20$; Vitamins ( 0.2 mg of thiamine, HCL; 1 µg of biotin; 1µg of $B_{12}$) Trace metal (0.0196 mg of $CuSO_4$, $5H_2O$, 0.044 mg of $ZnSO_4$, $7H_2O$; 0.02 mg of $CoCl_2$, $6H_2O$; 0.360 mg $MnCl_2$, $4H_2O$; 0.0126 mg of $Na_2MoO_4$, $2H_2O$); Sea water (to 1 liter) | Guillard, R.R.L. and J.H. Ryther. (1962) Studies on marine planktonic diatoms I, Cyclotella nana Hustedt and Detonula confervacae (Cleve) Gran. Can. J. Microbi~8: 229-239. |

| | | |
|---|---|---|
| **Modified F/2** | 27 g/L of NaCl;5.6 of $MgCl_2$,$6H_2O$;1.12 g/L of $CaCl_2$; 1 g/L of $KNO_3$; 0.07 g/L of $KH_2PO_4$; 0.04 g/L of $NaHCO_3$, 3.22 g/L of $MgSO_4$, $H_2O$; 0.075 g/L of $NaNO_3$, 0.005 g/L of $NaH_2PO_4$, Metal Solution and Vitamin Solution. | Guillard RRL (1975) Culture of Phytoplankton for Feeding Marine Invertebrates. In: Smith WL, Chanley MH (eds) Culture of Marine Invertebrate Animals: Proceedings — 1st Conference on Culture of Marine Invertebrate Animals Greenport. Springer US, Boston, MA, pp 29–60 |
| **BG-11 medium** | 1.5 g/L of $NaNO_3$; 0.04g/L of $K_2HPO_4$, $3H_2O$; 0.075 g/L of $MgSO_4$, $7H_2O$; 0.002 g/L of $Na_2CO_3$; 0.036g/L of $CaCl_2$, $2H_2O$; 0.058 g/L of $Na_2SiO_3$, $9H_2O$; 0.001 g/L of EDTA; 0.006g/L of citric acid; 0.006 g/L of Fe citrate and 1 ml of metal solution prepared in pure water and containing 2.86 g/L of $H_3BO_4$; 1.81 g/L of $MnCl_2$,$4H_2O$; 0.222 g/L $ZnSO_4$, $7H_2O$; 0.391 g/L $Nn_3MoO$,$2H_2O$; 0.079 g/L of $CuSO_4$, $5H_2O$; 0.0494 g/L of $Co(NO_3)_2$, $6H_2O$ | Allen MM (1968) SIMPLE CONDITIONS FOR GROWTH OF UNICELLULAR BLUE-GREEN ALGAE ON PLATES. J Phycol 4:1–4. https://doi.org/10.1111/j.1529-8817.1968.tb04667.x |

**Table S2**. Partial sequence of 18S rRNA gene of the isolated *Rhodomonas* sp.

| **18S partial sequence** |
| --- |
| >ATTCAAAATCCAGGATATCGTTTATTTGACGGTCCAAAAAACTACATGGATAACCGTAGTATTCTACTGCTA<br>ATACATGCCCAAAGGTCCGACTTACGAAGGACTGTATTTATTAGATTCCAAGCCACATTTCTGGTGATTCATA<br>ATAACTTCTCGAACCACAGCCCTCGCGGTGGTGGTGATTCATTCAAATTTCTGCCCTATCAACTTTCGATGGT<br>AGGATAGAGGCCTACCATGGTTTTAACGGGTGACGGAAAATTAGGGTTCGATTCCGGAGAGGGAGCCTGA<br>AAGACGGCTACCACATCCAAGGAAGGCAGCAGGCGCGCAATTACCCAATCCCGACTCGGGGGAGGTAGTG<br>ACAATAAATAACATCCGGGGCTAACCCCTTGTTATTGGATGACACCATTTAAATCCCTTTCCGAGAACAATTA<br>AGGGCAAGTCTGGTGCCGCACCCGCGGAATCCCGCTCCAAAAGCGTATATAAAGTTGTCGCATTAAAAACT<br>CGTATCCGATGCCGGCTCGGGAGGTGCCGCCTTGGTCGAAGGCTGCCGGTCCTATCTCCTGGGATCCTATC<br>ACTTAACTGAGGAAGGGACCAGGCTTTCTTTGAAAATTAAATGTCAGCGGCCACCTGGACCTACCTGGAAA<br>GGAAAGATTTTGTCTATTGTGTTGGGCCCAATAGTAACGGACGTTGGCCTATTGTGTGCAAGTAATCTGATA<br>CGAAAACTTCGCAACTCCGAGGTTCTAGTACACATGATGGTAGAGAACATCCTGAGTATCATAATGGGCAA<br>GATAGGACCTGGGGCCTGGGCGTCGAACCCATTTGTGGTCCTAGTAACTAGCACATAA |
